# Climate risks in urban areas

**DOI:** 10.2471/BLT.24.020224

**Published:** 2024-02-01

## Abstract

Engaging with communities in informal settlements presents opportunities to mitigate the health impacts of climate change, but government investment is also needed. Gary Humphreys reports.

Zilire Luka was asleep in bed when Cyclone Gombe hit the Malawi capital, Lilongwe. “It was raining hard, but it was the phone that woke me up,” says the executive director of the Center for Community Organization & Development (CCODE), a nongovernmental organization (NGO) based in the city.

The call came from a community leader in one of the informal settlements on the city’s outskirts. “He was reaching out for help,” says Luka. “He told me that bridges and roads were being washed away and people’s homes were collapsing. It was complete chaos.”

Cyclone Gombe hit Malawi on 11 March 2022 and followed two extreme weather events – Tropical Storm Ana and Tropical Depression Dumako – that had struck the country six weeks earlier.

As Luka points out, the 740 000 people living in Lilongwe’s slums (roughly 62% of the city’s 1.2 million population) were not the only ones to be impacted, but they were the most exposed, living in unregulated, unsafe buildings with inadequate water, sanitation and hygiene (WASH) infrastructure.

For Kate Medlicott, a water, sanitation and hygiene expert at the World Health Organization (WHO), the scenario is all too familiar. “People living in informal settlements tend to see the worst outcomes, especially those living in urban floodplains without adequate WASH systems or services,” she says.

According to Medlicott, around one third of the estimated 4.5 billion urban dwellers worldwide use pit toilets or septic tanks, which are normally the first to be overwhelmed in floods, spreading excrement and the pathogens it carries into the environment. “To make matters worse, overflowing sewers also drain into low-lying communities,” she says.

“We have come to see that what we’re building is socioeconomic equity.”

Margaret Mengo

The inevitable consequence is an increase in outbreaks of waterborne diseases, including cholera. Malawi is a case in point – inadequate water, sanitation and hygiene infrastructure having been identified as a key driver in the country’s biggest cholera outbreak since records began. The outbreak was first declared in March 2022 and, by October 2023, had killed 1768 of the 59 013 people reported to have been infected.

Medlicott is concerned that climate change will only exacerbate the challenges faced in cities. This is a view shared by Mathias Spaliviero, senior human settlements officer at the United Nations Human Settlements Programme (UN-Habitat).

“Climate-related flooding is the most recurrent hazard affecting cities, often exposing uncontrolled urban sprawl which reduces soil infiltration and increases runoff, as well as long-standing challenges such as urban poverty, the lack of affordable housing and inadequate infrastructure,” Spaliviero says.

Since 2015, UN-Habitat has been working with governments and partners to achieve sustainable development goal (SDG) 11 (make cities and human settlements inclusive, safe, resilient and sustainable). Like WHO, UN-Habitat advocates a comprehensive multi-pronged approach to addressing the problem, starting with ensuring robust governance, policy, planning and regulation.

However, as Spaliviero points out, getting governments to adopt and then implement an improved governance and policy framework is a challenge. “Unfortunately, cities are growing faster than governments can build the necessary institutional capacity to better plan and manage urban growth,” he says.

Moreover, governments in the countries most affected by the twin burden of climate change risk and informal development, generally lack the resources required to invest in urban infrastructure and housing, or have chosen to prioritize other investments.

As a result, progress on SDG 11, and particularly target 11.1 (by 2030, ensure access for all to adequate, safe and affordable housing and basic services and upgrade slums) has been mixed. “According to the latest UN reporting, slum populations are on the rise,” says Spaliviero, pointing out that the 1.1 billion people currently living in them is projected by the UN to rise by another 2 billion in the next 30 years.

So what is to be done? For Spaliviero, in the absence of progress on urban investment, planning and management, one way of addressing inequitable exposure to climate risk is to engage with the communities most affected.

This is a conviction founded on twenty years’ experience in the field and one core observation: people in informal settlements are motivated to change them.

Qudsia Huda, a health emergency expert at WHO, takes a similar view, adding that not only are community members motivated to initiate risk mitigation measures, they also typically have the best understanding of their surroundings, including the makeup of the local population, areas most likely to flood, etc.

This has implications for risk mitigation as well as emergency response. “Community members often have information about vulnerable groups such as pregnant mothers, children, the disabled,” Huda says. They can therefore be of tremendous assistance in directing health services where they are needed.”

While stressing the need for government engagement and investment, WHO has long supported community-based initiatives as part of disaster risk management, seeing them as an effective way to empower and encourage community members to participate in identifying problems and implementing needed interventions based on local needs, capacities, and available local resources.

While advocating for greater community engagement, Spaliviero is quick to point out obstacles that include the reluctance of communities to participate. “People see government officials coming into the slum and they think they are going to be moved,” he says, adding that local authorities also tend to disregard local communities as “incapable of planning and action.”

That such obstacles can be overcome is borne out by the experience of Habitat for Humanity, an NGO that works on community-led projects in countries worldwide, including in Malawi, applying an approach designed to encourage community engagement.

“[Money for climate and health] tends not to trickle down to where it is needed.”

*Mathias Spaliviero*

Developed by the International Federation of Red Cross and Red Crescent Societies and termed the Participatory approach for safe shelter awareness (PASSA), the approach involves an eight-step assessment of the community that includes the drafting of a timeline registering key events including disasters, and the generation of a baseline map that reflects the location of the houses and community infrastructure.

“PASSA is used to create an action plan to increase shelter safety,” explains Margaret Mengo, director of Habitat for Humanity’s program operations in Africa. However, as Mengo is keen to stress, it goes beyond that, notably by mapping residents’ occupations, the base assumption being that climate resilience is at least in part a question of economic resilience. “We’re builders but we have come to see that what we’re building is socioeconomic equity,” says Mengo.

In Lilongwe, CCODE is taking a similarly cross-sectoral approach, working in collaboration with the Federation of the Rural and Urban Poor (FRUP), a community finance initiative. Since 2003, the two entities have been leading an initiative known as the Malawi Alliance that is designed to encourage informal settlement communities to work together with NGOs and local government.

The Alliance is designed to raise awareness about climate-related risks such as flooding, while also supporting community-led monitoring and evaluation and strengthening local infrastructure, notably drainage.

According to Luka, community-led data collection and analysis is used to map and identify areas of high risk, drawing on approaches developed by Slum Dwellers International (SDI), a network of community-based organizations and federations of the urban poor that uses digital technology, including global positioning systems (GPS) technology.

“The mapping has made a huge difference,” says Luka, “helping us to highlight, for example, the vulnerability of certain houses, and the best places to site drainage capable of coping with flooding during storms.”

The data have also been used to inform discussions with representatives from Lilongwe City Council and other stakeholders which have led to the production of community resilience plans and risk management frameworks. 

The Alliance has also encouraged a sense of agency on the part of people living in the settlements, and encouraged women in the settlements to establish savings groups, which pool resources available while acting as a platform for community members to discuss and exchange ideas on how to achieve resilience. 

Spaliviero highlights obstacles; “There is money for climate and health, but partly because of the way finance is set up, fiduciary considerations and the need to produce onerous dossiers, it tends not to trickle down to where it is needed,” he says.

WHO’s Medlicott concurs; “The roots of these problems often lie outside the affected communities and are beyond their means to solve, including things like piped water and sewer infrastructure,” she says. “So, increasing government investment continues to be a priority.”

**Figure:**
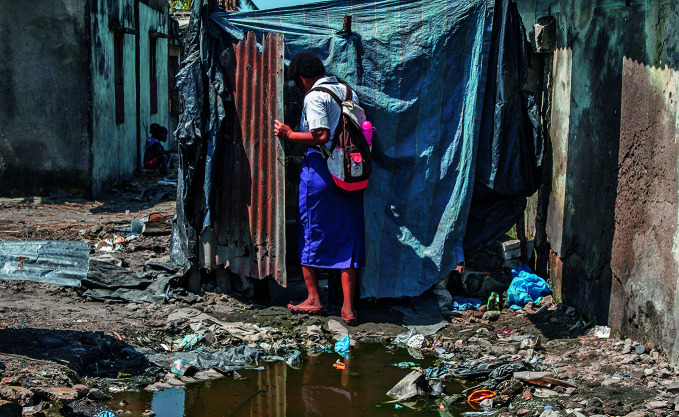
A woman enters her home in the Munhava district of Beira, Mozambique

*Médecins Sans Frontières*/Pablo Garrigos

**Figure:**
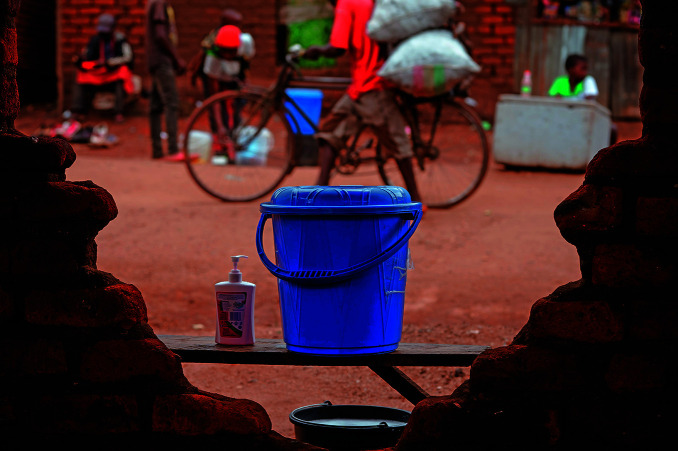
A hand-washing facility set up in Lilongwe, Malawi, as part of cholera response

WHO

